# Whole liver phase‐based R2 mapping in liver iron overload within a breath‐hold

**DOI:** 10.1002/mrm.30461

**Published:** 2025-02-18

**Authors:** Daiki Tamada, Ruvini Navaratna, Jayse Merle Weaver, Diego Hernando, Scott B. Reeder

**Affiliations:** ^1^ Department of Radiology University of Wisconsin‐Madison Madison Wisconsin USA; ^2^ Department of Medical Physics University of Wisconsin‐Madison Madison Wisconsin USA; ^3^ Department of Biomedical Engineering University of Wisconsin‐Madison Madison Wisconsin USA; ^4^ Department of Medicine University of Wisconsin‐Madison Madison Wisconsin USA; ^5^ Department of Emergency Medicine University of Wisconsin‐Madison Madison Wisconsin USA

**Keywords:** iron overload, liver iron concentration (LIC), quantitative imaging biomarkers, R_2_, relaxometry, T_2_

## Abstract

**Purpose:**

Diagnosis and treatment monitoring of iron overload increasingly relies on non‐invasive MRI‐based measurement of liver iron concentration (LIC). Liver R_2_ mapping is known to correlate well with LIC. However, traditional spin‐echo based R_2_ mapping methods have drawbacks such as long acquisition times and limited volumetric coverage. In this work, we present an optimized phase‐based R_2_ mapping method to quantify whole‐liver R_2_ in iron overload patients within a single breathhold.

**Theory and Methods:**

A recently developed phase‐based R_2_ mapping method is optimized in this study to improve estimation of high R_2_ values using reduced TR, spatial averaging, and R_1_ correction. Using Bloch equation simulations, the proposed optimization method was evaluated. Furthermore, the impact of fat on R_2_ bias was investigated through simulations. The feasibility of the optimized phase‐based R_2_ method was assessed in healthy volunteers and patients with iron overload and compared to reference STEAM‐MRS R_2_ measurements.

**Results:**

Simulations demonstrate that a shorter TR extends the dynamic range of R_2_ estimation to higher values and that averaging of signal phase before R_2_ estimation is necessary when R_2_ is high. Phantom experiments also demonstrate reduced phase‐based R_2_ bias using R_1_ correction. Good agreement (1.5 T: r^2^ = 0.76, 3.0 T: r^2^ = 0.70) between the modified phase‐based R_2_ method and reference STEAM R_2_ was found in healthy volunteers and iron overload patients over a wide range of LIC values.

**Conclusion:**

This study demonstrates the feasibility of the proposed phase‐based R_2_ method to accurately measure whole‐liver R_2_ mapping in severe iron overloaded patients during a single breathhold.

## 1 INTRODUCTION

Excess accumulation of iron in the body, or systemic iron overload, is toxic and can lead to complications such as cirrhosis, hepatocellular carcinoma, liver failure, pancreatic dysfunction, and heart failure, among other complications.^1^, ^2^, ^3^ Liver iron concentration (LIC) is well‐established as a surrogate for total body iron.^4^ Early detection, staging and treatment monitoring relies on accurate assessment of LIC.^5^, ^6^ LIC has been assessed historically using liver biopsy. However, biopsy is prone to sampling variability^2^, ^7^, ^8^ and is contraindicated in patients with bleeding diatheses (e.g. thrombocytopenia), which are common in many patients with iron overload. For these reasons, it is no longer common to use biopsy to assess LIC, and non‐invasive methods such as MRI are preferred.

Non‐invasive MR relaxometry techniques, particularly R_2_ and R_2_* mapping, have been calibrated to LIC through biopsy‐MRI correlation studies.^9^, ^10^ Although R_2_* mapping, derived from multi‐echo gradient‐echo sequences, offers faster acquisition times,^11^ R_2_ mapping has demonstrated comparable accuracy in estimating LIC.^10^ Additionally, R_2_ mapping exhibits reduced sensitivity to susceptibility effects and image voxel size,^12^ potentially providing more robust measurements in certain clinical scenarios. R_2_ measurements may also offer complementary information to R_2_*, because they have different dependencies on microscopic iron distribution and molecular diffusion within the tissue.^13^ However, current R_2_ mapping methods, such as spin‐echo (SE) R_2_‐based mapping, suffer from excessive acquisition times (˜20 min) and limited spatial coverage of the liver.^6^ There is an unmet need for an R_2_‐based iron quantification method that provides whole‐liver, rapid iron concentration measurement over a wide range of LIC.

Recent advances have introduced several promising R_2_ mapping techniques for liver imaging. For example, Bieri et al.^14^ developed a 3D R_2_ mapping method using partial spoiled steady state free precession, enabling rapid R_2_ mapping within a short timeframe. Simultaneous R_1_ and R_2_ mapping methods using preparation pulses have also shown potential, facilitating free‐breathing quantitative liver imaging.^15^, ^16^ Although these methods hold potential for liver applications, their sensitivity to high R_2_ values may be constrained by the relatively lengthy effective echo times used.

To address this limitation, a phase‐based (PB) R_2_ mapping method has been proposed, allowing for whole‐liver R_2_ measurements within a single breathhold.^17^ In this method, R_2_ relaxation is encoded directly into the phase of an RF phase‐modulated 3D gradient echo (GRE), allowing for rapid estimation of whole‐liver R_2_. Because PB R_2_ mapping enables short TR and TE, it is promising to measure the liver with high R_2_. Although the feasibility of this method has been demonstrated in healthy volunteers at 3.0 T, significant modifications of this method are needed to quantify R_2_ in tissues with high iron concentration such as those seen in the liver of patients with iron overload. The purpose of this work is to demonstrate the feasibility of a PB R_2_ mapping method for single‐breathhold, whole‐liver volumetric R_2_ quantification in patients with iron overload at both 1.5 and 3.0 T.

## 2 THEORY

In previous work,^17^ it was shown that the signal phase of a GRE acquisition increases monotonically with increasing T_2_ (thereby decreasing monotonically with increasing R_2_) when the phase of the RF excitation is modulated using quadratically increasing phase with small increments. Using a lookup table generated through recursive equations,^18^, ^19^ R_2_ (=1/T_2_) can be estimated from the signal phase. Although the method is relatively insensitive to tissue R1 (=1/T_1_), quantification of R_2_ using this method requires knowledge of, or reasonable assumptions of tissue R_1_, along with the known TR, flip angle, and RF phase increment of the acquisition. Although the feasibility of this method to rapidly quantify whole‐liver T_2_ has been demonstrated in healthy volunteers without iron overload, significant modifications are needed to estimate high liver R_2_ (low T_2_) in patients with iron overload. In this work, we propose three modifications needed to enable PB R_2_ mapping method in the presence of high R_2_: (1) reduced TR; (2) spatial averaging; and (3) R_1_ correction. These modifications are described in detail below.

### 2.1 Signal representation

We consider a sequence with an RF pulse characterized by flip angle of α, RF phase (ϕ), and repetition time TR.^18^ RF phase modulation is performed by incrementing ϕ quadratically such that ϕ(n)=ϕ(n−1)+nθ, where θ is the RF phase increment. In this case, the steady‐state complex signal (S_0_) after RF excitation can be expressed by the following equation:

(1) $$S_{0}\left(R_{1},R_{2}\right)=\beta \eta e^{-R_{1}\mathrm{TR}}+\beta \left[\eta^{2}-\epsilon \left(e^{-R_{2}\mathrm{TR}}-\epsilon \right)\right],$$

where β, η, and ϵ are coefficients that depend on TR, R_1_, R_2_, α, and θ can be derived by solving recursive equations proposed by Sobol et al.^18^, ^19^

In our previous study, using Eq. (1), we demonstrated that T_1_‐ and T_2_‐weighted signals are encoded as the magnitudes of the imaginary and real components of the GRE signal, respectively. In other words, these imaginary and real components correspond to free induction decay (FID)‐ and echo‐like signals, which are similar to SSFP‐FID and SSFP‐Echo in dual‐echo steady‐state imaging.^20^, ^21^ Consequently, the signal phase should theoretically range from 0° to 90°.

### 2.2 R_2_ estimation

Using the proposed analytical equation, rather than Bloch equation simulation, allows for rapid calculation of the GRE signal used to generate the lookup table. The lookup table contains values of the steady‐state signal phase across practical ranges of R_1_ and R_2_, using fixed sequence parameters (see Subsection 3.8).

R_2_ can be estimated by finding the value of R_2_ that best matches the signal phase from the lookup table with the measured phase of signal $S_{\mathrm{acq}}^{*}$, as described by:

(2) $$R_{2}\left(R_{1}\right)=\underset{R_{2}}{\text{argmin}}\left|∠\left(S_{0}\left(R_{1},R_{2}\right)\cdot S_{\mathrm{acq}}^{*}\right)\right|.$$

In our previous work, a fixed T_1_ value was used for R_2_ estimation.^17^ The previous work has demonstrated that PB R_2_ mapping is relatively insensitive to R_1_, although approximate knowledge of or assumption of the R_1_ of tissue is necessary to generate an accurate lookup table.

### 2.3 Fat‐related bias

Fat accumulation in the liver can also introduce bias in PB R_2_ mapping. This poses a challenge as fat has a shorter T_1_ than water, leading to stronger signal and potential confounding factor. In patients with high iron overload, fat will also exhibit longer T_2_ than water, further confounding the acquired signal phase.

Assuming presence of fat with six spectral peaks, the signal $S_{f}$ from tissue containing both water and fat components can be explained as

(3) $$S_{f}\left(\;R_{1},R_{2}\right)=\left(\rho_{W}\left(R_{1},R_{2}\right)+\rho_{F}\left(R_{1},R_{2}\right)\sum_{p=1}^{P}u_{p}e^{j2\pi f_{p}\mathrm{TE}}\right),$$

where $\rho_{W}$ and $\rho_{F}$ are signal amplitude for water and fat, and $u_{p}$ and $f_{p}$ are amplitudes and frequencies for the spectral peaks, and TE is echo time. The values for $u_{p}$ and $f_{p}$ were determined based on a previous study.^22^ This equation demonstrates two concepts: (1) the presence of fat signal results in additional phase, leading to bias in R_2_ estimation, and (2) an increase in the relative signal magnitude of fat can also lead to an increased phase error. The significance of the bias in R_2_ measurement because of fat will be investigated further in subsequent sections (see Sections 3.5 and 4.4).

### 2.4 Essential improvements for high R_2_ measurements

In cases of iron‐overloaded patients, it is essential to modify the conventional PB R_2_ mapping because of low SNR and bias because of R_1_, as described below.

TR plays an important role for determining effective TE of the real component signal to maximize the dynamic range of the signal phase. Although the echoes in GRE sequences are complex because of the many signal pathways involved,^23^ the shortest effective TE of one of the major pathways is 2 × TR,^24^ which provides a general guideline for selecting the appropriate TR for the sequence. Therefore, a shorter TR is needed for R_2_ measurement in tissues with high R_2_ values (see Subsection 3.1).

In cases of high R_2_, the signal becomes weaker because of strong T_2_ and T_2_* decay. Consequently, the signal phase is more susceptible to noise, including voxels with non‐physical negative phase values that would lead to estimation of high R_2_ values. Ensuring a sufficient SNR is crucial to mitigate the possibility of non‐physical negative phase values. In this study, we applied spatial averaging as described in Subsection 3.2.

Although the approach explained in Subsection 2.2 provides a reasonable R_2_ in most cases, additional assumptions will be used in this study for iron‐overload cases, as explained in Subsection 3.3. Although R_1_ is relatively low (e.g., 1.2 s^−1^ at 3.0 T)^25^, ^26^ and constant throughout normal liver, R_1_ is known to increase in the presence of iron.^27^ Therefore, bias in R_2_ estimation in the presence of iron overload is anticipated if iron‐related changes in R_1_ are not considered.

## 3 METHODS

This section describes the methodological approach for whole‐liver PB R_2_ mapping in the assessment of liver iron overload. This includes spatial averaging, R_1_ correction, reconstruction methods, simulations for optimizing TR, flip angle, and RF phase increment, evaluating fat‐induced bias, conducting phantom experiments, and performing in vivo human studies. Finally, we describe the analysis procedures for R_2_ maps. All of the calculation was implemented using MATLAB 2022b (The MathWorks).

### 3.1 TR optimization

TR was determined based on expected TE of the tissues. In patients with iron overload, LIC values have been measured as high as 30 to 40 mg Fe/g dry tissue, corresponding to R_2_ values at 1.5 T of 250 to 300 s^−1^ (T_2_ = 3–4 ms).^9^ This necessitates short TR values on the order of 3 to 4 ms at 1.5 T. Using the approximation R_2_ (3.0 T) ≈ 1.5 × R_2_ (1.5 T),^28^ this translates to the highest observed R_2_ at 3.0 T being approximately 300 to 450 s^−1^ (T_2_ = 2–3 ms). Therefore, we anticipate that a TR on the order of 3 to 4 ms at 1.5 T and 2 to 3 ms at 3.0 T is necessary for accurate R_2_ estimation in patients with severe iron overload, ensuring the TE is short enough compared to the effective TE.

We validate the effect of TR on R_2_ estimation using noiseless Bloch equation simulations of the PB R_2_ encoding method. Simulations were performed using 1000 isochromats with flip angle = 18°, RF phase increment = ±2, and R_1_ = 1 s^−1^, as described in the Theory section. The signal phase as a function of R2 (1–500 s^−1^) was plotted for various TR values (3, 6, and 9 ms) for these noiseless simulations. To achieve shorter TR for better sensitivity to the high R_2_ range, fat suppression techniques such as chemical shift‐encoded (CSE)‐MRI and water‐only excitation^29^ were not included in this study. This approach also helps minimize TE, thereby reducing the impact of T_2_* decay on the signal. Further, in the presence of severe iron overload, line‐width broadening of water and fat resonance peaks renders CSE‐MRI method ineffective at separating water and fat signals.^30^, ^31^

### 3.2 Spatial averaging

To mitigate possible R_2_ bias resulting from negative phase values under severe noise conditions, we applied spatial averaging to the acquired complex images before R_2_ mapping calculations. Specifically, we used a 2D rectangular averaging kernel with the size of 3 × 3, where each element of the kernel has a weight of 1/9 to ensure signal normalization after filtering.

### 3.3 R_1_ ‐corrected R_2_ mapping

To estimate R_1_‐corrected R_2_ mapping, we propose using the known relationship between R_1_ and R_2_ in patients with iron overload. This relationship can be derived from the results of a recent multi‐center study,^27^ where:

(4) $$R_{1c}\left(R2\right)=\left\{\begin{array}{ll} 6.5\times 10^{3}\cdot R_{2}+1.23 & \text{when}\;B_{0}=1.5\;T\\ 3.8\times 10^{3}\cdot R_{2}+0.89 & \text{when}\;B_{0}=3.0\;T \end{array}\right.,$$

with units of s^−1^. The steady‐state complex signal $S\prime_{0}$ with R_1_‐R_2_ calibration can be derived by substituting Eq. (4) for R_1_ in Eq. (1), which is used for generating the lookup table as;

(5) $$S_{0}^{\prime }\left(R_{2}\right)=\beta \eta e^{-R_{1c}\left(R2\right)\mathrm{TR}}+\beta \left[\eta^{2}-\epsilon \left(e^{-R_{2}\mathrm{TR}}-\epsilon \right)\right].$$

R_1_‐corrected R_2_ ($\hat{R_{2}}$) can be estimated similarly to Eq. (2) by

(6) $$\hat{R_{2}}=\underset{R_{2}}{\text{argmin}}\left|∠\left(S\prime_{0}\left(R_{2}\right)\cdot S_{\mathrm{acq}}^{*}\right)\right|.$$

We note that this method also produces an estimate of the R_1_ ($\hat{R1}$) of the tissue using the following equation, although this is not the primary purpose of this work.

(7) $$\hat{R1}=R_{1c}\left(\hat{R_{2}}\right)$$

The R_2_ value for each voxel was then estimated from the phase of the images using a pre‐calculated lookup table as the following subsection.

### 3.4 Flip angle and RF phase increment optimization

Optimization of the flip angle and RF phase increment that maximizes the performance of R_2_ estimation in the presence of iron overload is needed. Simulations were performed to select a flip angle and phase increment that allows for R_2_ estimation that is relatively insensitive to B_1_ inhomogeneity and noise effects. Bloch equation simulations of the PB R_2_ method were performed using 1000 isochromats with R_1_ = 1 s^−1^ with TR = 3 ms, a typical minimum TR on high‐performance MR systems. This was performed over a range of flip angles,^4^, ^5^, ^6^, ^7^, ^8^, ^9^, ^10^, ^11^, ^12^, ^13^, ^14^, ^15^, ^16^, ^17^, ^18^, ^19^, ^20^, ^21^, ^22^, ^23^, ^24^ RF phase increments,^1^, ^2^, ^3^, ^4^, ^5^ and R_2_ values observed in iron overloaded tissue at both 1.5 T and 3.0 T,^9^ of 50 to 450 s^−1^.

To find an optimal flip angle/phase increment combination in the presence of B_1_ inhomogeneity, noiseless simulations were performed over the same flip angles and phase increments with variable B_1_ calibration coefficient^32^ of −1.2. The range for was chosen based on the lower and upper limits of B_1_ inhomogeneities in the liver at 3.0 T according to Roberts et al.^32^ The normalized absolute R_2_ bias $\left(\frac{\left|\mathrm{Bia}s_{R2}\right|}{R2}\right)$ was calculated for each combination. To determine the worst‐case bias performance of each flip angle/phase increment combination, we calculated the maximum normalized absolute R_2_ bias across all R_2_ and γ values.

To find an optimal flip angle/phase increment combination under noisy conditions, Gaussian complex noise (SN≈20) was added to the signal. The normalized SD on estimated R_2_ $\left(\frac{\sigma_{R2}}{R2}\right)$ was calculated for each combination. To determine the worst‐case performance, we calculated the maximum normalized SD across all R_2_ values.

### 3.5 Fat bias simulation

To evaluate the R_2_ measurement bias introduced by fat and determine exclusion criteria for in vivo experiments, we conducted simulation studies. GRE signals were simulated using Eq. (7) with optimized parameters (TR (1.5 T/3.0 T) = 3.2/3.1 ms, flip angle = 18, RF phase increment = 1.5) determined based on acquisition parameters described in Sections 3.1 and 3.2, and varying proton density fat fraction (PDFF) values ranging from 0% to 30% and R_2_ values from 1 to 300 s^−1^ at 1.5 and 3.0 T. We estimated R_2_ using Eq. (2) for each combination of PDFF and R_2_ from these calculated signals. From these estimations we computed the measurement bias ($100\times \frac{\left|{\hat{R}}_{2}-R_{2}\right|}{R_{2}}$ [%]), where ${\hat{R}}_{2}$ is the estimated R_2_.

### 3.6 Phantom experiments

Eleven agarose gel (2% w/v) vials with varying MnCl_2_ concentration (0.3–3.7 mM) were constructed to achieve high R_2_ values, nominally 50 to 300 s^−1^ at 3.0 T, and high R_1_ values, nominally 4 to 30s^−1^. 25 mL vials were imaged on a 1.5 T MR system (Signa Artist, GE Healthcare) using a standard head/neck coil (Head Neck Unit, 16 channels, GE Healthcare) and a 3.0 T MR system (Signa Premier, GE Healthcare) using a standard head coil (AIR coil, 48 channel, GE Healthcare). Reference R_2_ and R_1_ values were found using a single‐echo SE or inversion‐recovered spin‐echo (SE‐IR) sequence, respectively. The reference R_1_ and R_2_ maps were used to determine the R_1_‐R_2_ calibration relationships $R_{1c}$. Signals were fit using least squares fitting to a mono‐exponential decay signal model in MATLAB to estimate R_2_ and a standard inversion recovery signal model to estimate R_1_ on a voxel‐by‐voxel basis. To estimate R_2_ of the phantom, 3D RF phase modulated GRE images, explained in Theory 2.1, were acquired. As described in the previous study,^17^ the sequence consists of two‐pass acquisition with opposite RF phase increment to remove background phase. Acquisition parameters are shown in Table 1. The flip angle and RF phase increment for the acquisition were determined based on the simulation described in Section 3.2.

**TABLE 1:** Acquisition parameters for the phantom experiments and volunteer/patient study.

	Phantom experiments			Volunteer/ patient study			
Pulse sequence	Single‐ echo SE	SE‐IR	Phase‐based GRE	Phase‐based GRE	CSE‐MRI	STEAM‐ MRS	Multi‐TR/TE STEAM‐MRS
TE (ms)	9, 9.5, 10, 12, 16, 24	10	(1.5 T) 1.0 or (3 T) 0.8	0.9	(1.5 T) TE_1_ = 0.9, ∆TE = 0.7 (3 T) TE_1_ = 0.8, ∆TE = 0.6 No. of echoes = 8	10, 13, 16, 19, 22	10 100, 250, 300, 500, 700, 900, 1100
TI (ms)	N/A	50, 100, 150, 200, 300	N/A	N/A	N/A	N/A	N/A
TR (ms)	2000	2000	(1.5 T) 3.4 or (3 T) 3.1	(1.5 T) 3.2 (3 T) 3.1	(1.5 T) 7.5 or (3 T) 6.2	3500	140–10 000 (29 TRs)
Flip angle (°)	N/A	N/A	18	18	(1.5 T) 10 or (3 T) 3	N/A	N/A
Phase increment (°)	N/A	N/A	±1.5	±1.5	N/A	N/A	N/A
Matrix	160 × 160	160 × 160	100 × 100 × 10	100 × 100 × 28	128 × 128 × 36	N/A	N/A
FOV	20 × 20 cm^2^	20 × 20 cm^2^	48 × 48 × 10 cm^3^	48 × 48 × 22 cm^3^	42 × 42 × 29 cm^3^	20 × 20 × 20 mm^3^	20 × 20 × 20 mm^3^
No. of slices	1	1	N/A	N/A	N/A	N/A	N/A
Slice thickness (mm)	10	10	10	8	8	20	20
ARC acceleration factor (PE × SL)	N/A	N/A	1	2 × 1	2 × 1.5	N/A	N/A
Scan time (s)	320	344	(1.5 T) 6.4 (3.0 T) 7.2	11	21	18	22

*Note*: All acquisitions were performed at either 1.5 T or 3.0 T. A low resolution (100 × 100) phase‐based GRE was acquired to achieve a very short TR (˜3.1 ms) for very high R_2_ estimation in patients if needed.

Abbreviations: CSE, chemical shift‐encoded; GRE, gradient echo; SE, spin echo; SE‐IR, inversion‐recovered spin‐echo; STEAM‐MRS, stimulated echo acquisition method magnetic resonance spectroscopy.

### 3.7 In vivo experiments

Six healthy volunteers participated in an in vivo feasibility study. In addition, subjects with known or suspected liver iron overload were recruited consecutively as part of an ongoing multi‐center liver iron study.^33^ All human imaging was performed under institutional review board approval after obtaining informed written consent. Liver imaging was performed on both a 1.5 T clinical MR system (Signa Artist, GE Healthcare) and a 3.0 T clinical MR system (Signa Premier, GE Healthcare) using a posterior and anterior receive array coil (AIR coil, GE Healthcare) for the abdomen.

Three dimensional RF phase modulated GRE images of the whole‐liver were acquired for PB R_2_ estimation. Sequence parameters are tabulated in Table 1. The same flip angle and RF phase increment were used as with phantom experiments. To provide a reference for liver R_2_ measurements, single‐voxel, multi‐TE STEAM‐MRS^34^ was acquired on a 20 × 20 × 20 mm^3^ volume in the right lobe of the liver, avoiding blood vessels. Importantly, STEAM‐MRS data were acquired in a single breathhold and, therefore, provide a reliable reference for R_2_, unconfounded by respiratory motion.^27^, ^35^, ^36^ In addition, multi‐TR/TE STEAM‐MRS data were acquired to obtain reference R_1_. The R_2_ and R_1_ of water and fat were estimated jointly from spectroscopy using non‐linear least‐squares fitting^37^ in MATLAB, with R_2_ and R_1_ of water used as a reference for in vivo R_2_ and R_1_ estimation.

Multi‐echo 3D CSE imaging were also acquired for R_2_* mapping using a commercial CSE‐MRI method (IDEAL IQ, GE Healthcare). R_2_* maps were generated automatically online.

To evaluate the repeatability of the PB R_2_ method, repeated PB R_2_ at 1.5 T and 3.0 T. After the initial PB acquisition, subjects were removed from the magnet, the anterior coil element removed, and the subject was asked to sit up. The subject and coil were then repositioned, localizer images repeated and the PB R_2_ acquisition was repeated.

Acquisition parameters are shown in Table 1. TR and flip angle were determined based on simulations in Subsections 3.1 and 3.4.

### 3.8 PB R_2_ Reconstruction

The R_2_ value for each voxel was estimated from the phase of the images using a pre‐calculated lookup table based on the analytical expressions of Sobol et al.^18^, ^19^ R_1_‐corrected R_2_ estimation was performed as described in the (Eqs. [5] and [6]). To assess the effect of R_1_ correction and averaging methods on R_2_ mapping, we also generated R_2_ maps without R_1_ correction or averaging. The reconstruction was performed as described in Subsection 2.2 of the Theory section (Eq. [2]), using fixed T_1_ values: 1000 ms for phantom experiments, and 576 ms at 1.5 T and 812 ms at 3.0 T for in vivo experiments. This lookup table covered a range of R_2_ values from 10 to 300 s^−1^. The sequence parameters, which were optimized as explained in the following subsections, outlined in Table 1 were used for these calculations.

### 3.9 Analysis

In phantoms, a 2 cm^2^ circular region of interest (ROI) was placed in the center of each vial on the central slice of the phase map to measure R_2_.

For in vivo imaging, a 7 cm^2^ liver ROI was placed on the phase map approximately co‐localized with the center of the STEAM‐MRS voxel. The locations of the in vivo ROIs were adjusted to avoid visible artifacts or vessels, if present.

In phantoms, linear regression was performed to compare PB R_2_ estimates with reference single‐echo SE R_2_ estimates. Similarly, linear regression was conducted for in vivo data with and without to assess the relationship between PB R_2_ estimates and the reference values. The Wilcoxon signed‐rank test was used to compare R_2_ values obtained with and without R_1_ correction and spatial averaging. Bland–Altman analysis was performed to compare PB R_2_ estimates and reference STEAM‐MRS estimates in in vivo imaging. Bias and 95% limits of agreement (LOA) were calculated. The intraclass correlation coefficient (ICC) was calculated for the repeated PB R_2_ measurements at 1.5 T and 3.0 T. To confirm the validity of R_1_‐R_2_ calibration, PB R_1_ was compared with R_1_ measured with the IR‐SE and multi‐TR/TE STEAM MRS.

## 4 RESULTS

### 4.1 Patient recruitment

We successfully recruited 38 adults with an average age of 53 years (range, 14–81 years), including 29 men and 9 women. We included 34 and 38 cases for analysis of R_2_ measurements acquired at 1.5 T and 3.0 T, respectively. The criterion for rejecting PB measurements because of high PDFF, which indicates a bias in R_2_ measurement exceeding 20%, was determined based on the simulations described in Sections 3.5 and 4.4. Four subjects imaged at 3.0 T were excluded because of high PDFF (3 cases) and failure of the STEAM‐MRS acquisition (1 case). For analysis of R_1_, we included 33 and 34 cases acquired at 1.5 T and 3.0 T, respectively. For R_1_ analysis, we included 33 cases at 1.5 T and 34 cases at 3.0 T. At 1.5 T, four cases were excluded: failure of the multi‐TR/TE STEAM‐MRS acquisition (2 cases) and failed data transfer (2 cases). At 3.0 T, five subjects were excluded: high PDFF (3 cases) and failure of the multi‐TR/TE STEAM‐MRS acquisition (2 cases). Overall, 17 and 13 patients underwent repeated scans at 1.5 T and 3.0 T, respectively.

### 4.2 TR optimization

**FIGURE 1:**
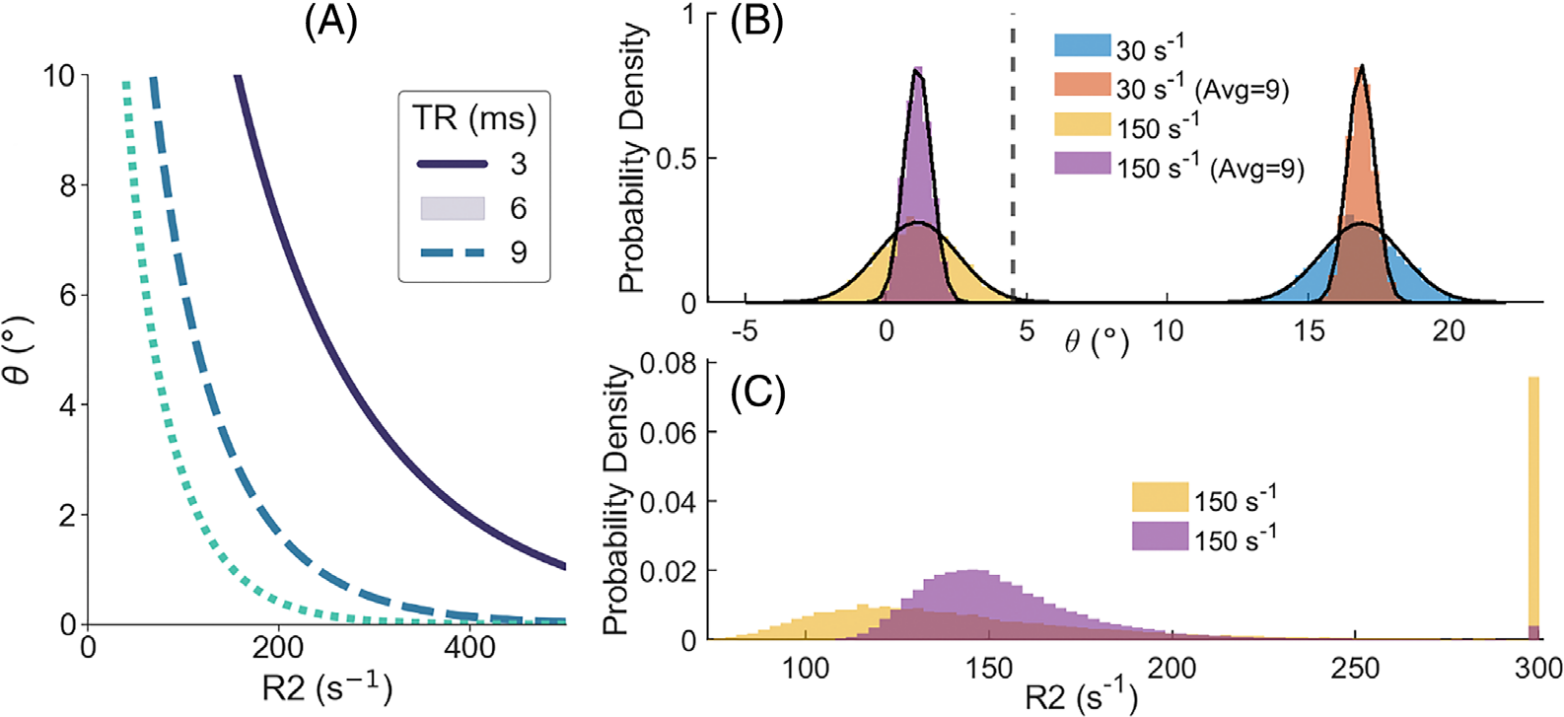
Simulations demonstrate the necessity of a reduced TR and the use of spatial averaging to improve estimation of high R_2_ values. (A) Signal phase (θ) as a function of R_2_ is plotted for various TRs in noiseless simulations. As TR increases, the signal phase of higher R_2_ values approaches zero, which can lead to non‐physical negative phase values because of noise. (B) The distribution of signal phase for various R_2_ is plotted in simulations with added noise. The phase distribution for R_2_ = 150 s^−1^, representing high iron tissue, contains negative values resulting in inaccurate R_2_ estimates if no averaging is done. (C) The distribution of R_2_ is skewed by noise, with non‐physical negative phase values interpreted as the highest R_2_ values in the lookup table.

As shown in Figure 1A, shorter TR enables higher R_2_ estimation. Using a TR of 3 ms and assuming we can reliably measure phase as low as 5, corresponds to a maximum measurable R_2_ of approximately 250 s^−1^. This R_2_ corresponds to a theoretical maximum measurable LIC of 32 mg/g at 1.5 T, and an LIC of 15 mg/g at 3.0 T according to the correlation by St. Pierre et al.^9^ Based on these results, we propose the use of the shortest possible TR for the PB R_2_ encoding method.

As shown in Figure 1B, the phase of the GRE signal may be negative in tissues with high R_2_ because of the presence of noise. Additionally, Figure 1C presents the distribution of the estimated R_2_ values corresponding to Figure 1B. The distribution of estimated R_2_ values is skewed by noise, and non‐physical negative phase values are interpreted as the highest R_2_ values in the lookup table. We have conducted additional experiments to investigate how TR impacts measurement bias and variability in Figure S1. These results suggest that the proposed spatial averaging approach can mitigate this bias.

### 4.3 Flip angle and RF phase increment optimization

**FIGURE 2:**
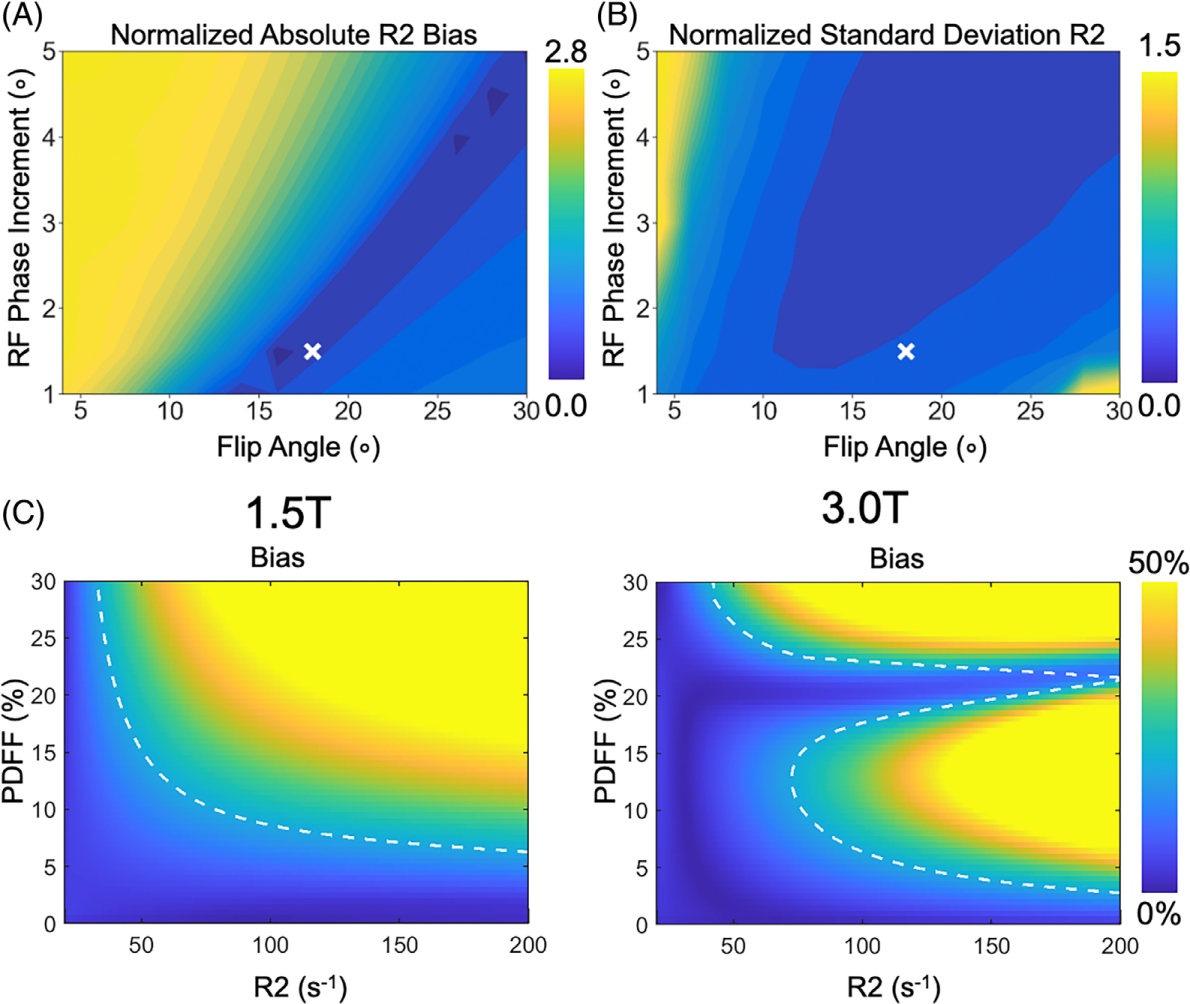
Simulations highlight optimal RF settings for minimizing R_2_ bias and noise, while revealing proton density fat fraction (PDFF)‐induced measurement bias, particularly at high R_2_ values and 3.0 T. (A) Simulations demonstrate low bias from B_1_ inhomogeneity and good noise behavior with a flip angle of 18° and RF phase increment of ±1.5°. (A) The maximum normalized absolute R_2_ bias (|*Bias*_*R2*_|/*R2*) across the wide range of R_2_ (50–450 s^−1^) and B_1_ inhomogeneity γ (0.6–1.2) as a function of flip angle and RF phase increment is plotted for noiseless simulations. (B) The maximum normalized SD of R_2_ (σ_*R2*_/*R2*) as a function of flip angle and RF phase increment is plotted for simulations with added noise. The chosen flip angle of 18° and RF phase increment of ±1.5° is marked with a green cross on each plot. (C) Simulation revealed the fat‐induced bias in R_2_ measurements using the phase‐based method, particularly in cases of high R_2_. The plots show the bias of R_2_ measurement as a function of R_2_ and PDFF at (A) 1.5 T and (B) 3.0 T. The white dashed lines indicate where the bias exceeds 20%. Because the T_1_ of fat is shorter than that of water, the signal magnitude of fat is higher than that of water. Consequently, phase errors can be caused by fat accumulation even when PDFF is small. The bias is more pronounced when R_2_ is high at 3.0 T compared to 1.5 T, as the T_1_ of water becomes much longer than that of fat.

As shown in Figure 2A,B, B_1_ sensitivity and noise performance are dependent on the choice of flip angle and RF phase increment. Figure 2A shows that specific combinations of flip angle and RF phase increment result in smaller R_2_ bias. In contrast, a relatively wide range of combinations of flip angle and RF phase increment is acceptable for achieving small measurement SD, as shown in Figure 2B, although a lower flip angle below 10° exhibits a high SD. Because higher RF flip angles can worsen the excitation slice profile, we used a flip angle of 18° and an RF phase increment of ±1.5°, marked with a white cross in Figure 2A,B. This combination provides low R_2_ bias because of B_1_ inhomogeneities and good noise performance with low R_2_ SD.

### 4.4 Fat bias simulation

As shown in Figure 2C, R_2_ is significantly biased in tissues with both high R_2_ and high fat content. In contrast, bias is minimal in tissues with lower R_2_, even in the presence of fat. The white dotted lines indicate where the bias exceeds 20%, which is exclusion criteria used for in vivo experiments. At 1.5 T, R_2_ measurement is relatively robust to fat accumulation, even when R_2_ is elevated. In contrast, at 3.0 T even small PDFF values lead to R_2_ bias when R_2_ is high. This difference is primarily attributed to the fact that the T_1_ relaxation time of water is shorter at 1.5 T compared to 3.0 T. Consequently, the relative fat signal is higher at 3.0 T because of longer T_1_ of water relative to fat at 3.0 T, leading to greater bias at 3.0 T. The off‐resonance frequencies of fat species differ between 1.5 T and 3.0 T, causing undesirable phase errors that contribute to bias in R_2_ estimation. As a result, the distribution of R_2_ bias differs significantly between these two field strengths.

### 4.5 Phantom experiments

From the obtained R_1_ and R_2_ maps measured using SE and SE‐IR, we determined the R_1_‐R_2_ calibration relationships as the following: $R_{1c}\left(R2\right)=0.14\cdot R_{2}+0.59$ at 1.5 T and $R_{1c}\left(R2\right)=9.2\times 10^{-2}\cdot R_{2}+1.3$ at 3.0 T.

**FIGURE 3:**
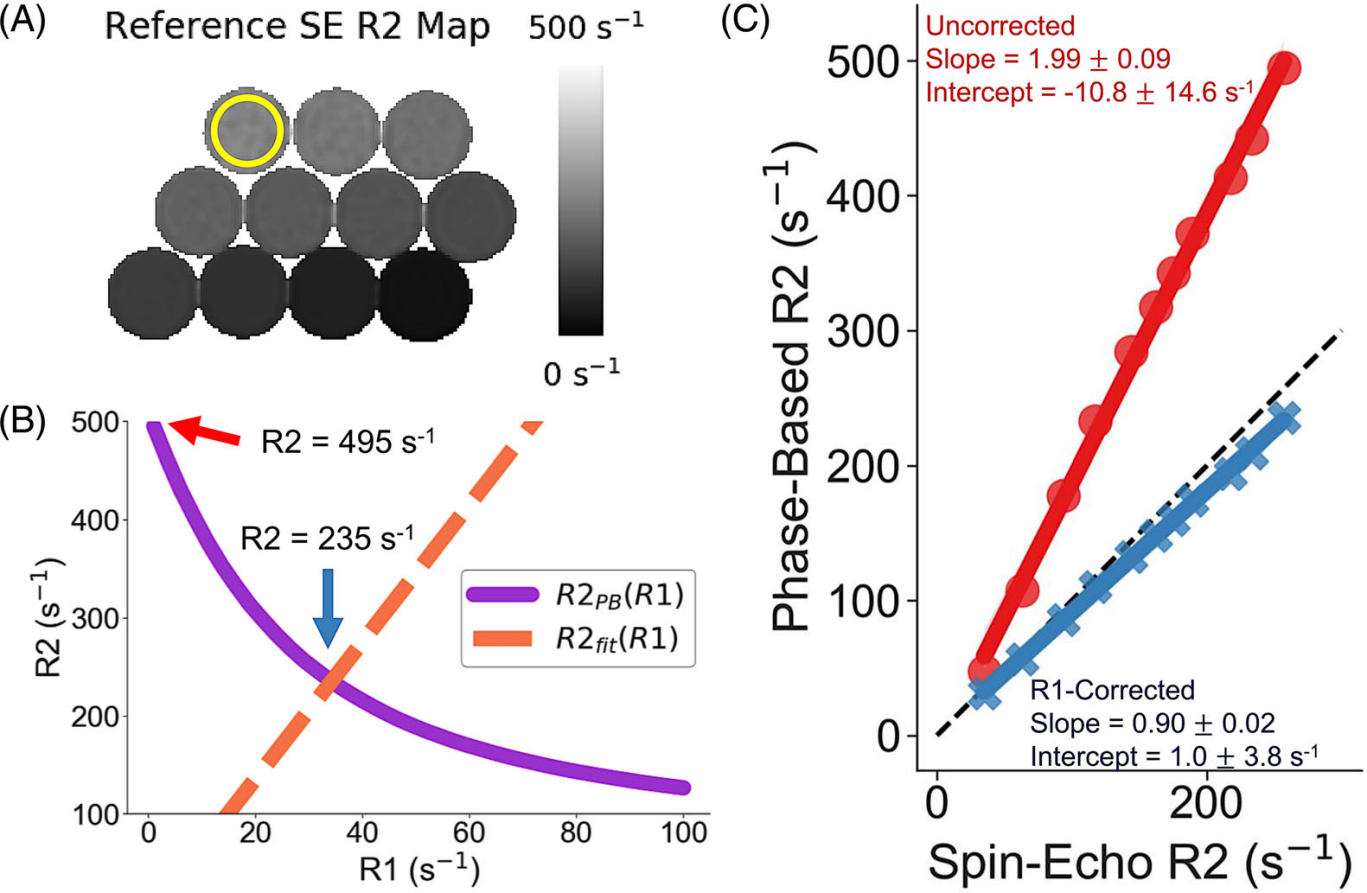
Phantom experiments demonstrate improved R_2_ estimation with R_1_ correction (Equations [5] and [6]). (A) Reference R_2_ map generated from a single‐echo spin‐echo (SE) sequence at 1.5 T. (B) An example of the uncorrected (red arrow) and corrected (blue arrow) R_2_ estimate are plotted for a single vial (yellow region of interest, reference R_2_ = 257 s^−1^). (C) Linear regressions of phase‐based R_2_ as a function of single‐echo SE R_2_ for the uncorrected (assuming R_1_ = 1 s^−1^) and R_1_‐corrected phase‐based R_2_ method are shown. Slope and intercept are shown with their 95% confidence interval. The identity line is plotted as a dashed line.

The phantom experiments were effective to demonstrate the accuracy of the proposed method, as shown in Figure 3. With a fixed assumption of R_1_ = 1 s^−1^ across all vials, the PB R_2_ estimation overestimated the reference SE R_2_ estimates, as shown by the red line in Figure 3C. However, after applying R_1_ correction, PB R_2_ measurements closely aligned with the reference, as shown by the blue line in Figure 3C. The purple line in Figure 3B also illustrates the relationship between R_1_ and R_2_ corresponding to the phase of a vial highlighted as the yellow ROI in Figure 3A. This demonstrates that R_2_ was uniquely determined using the prior knowledge of $R_{1c}$.

**FIGURE 4:**
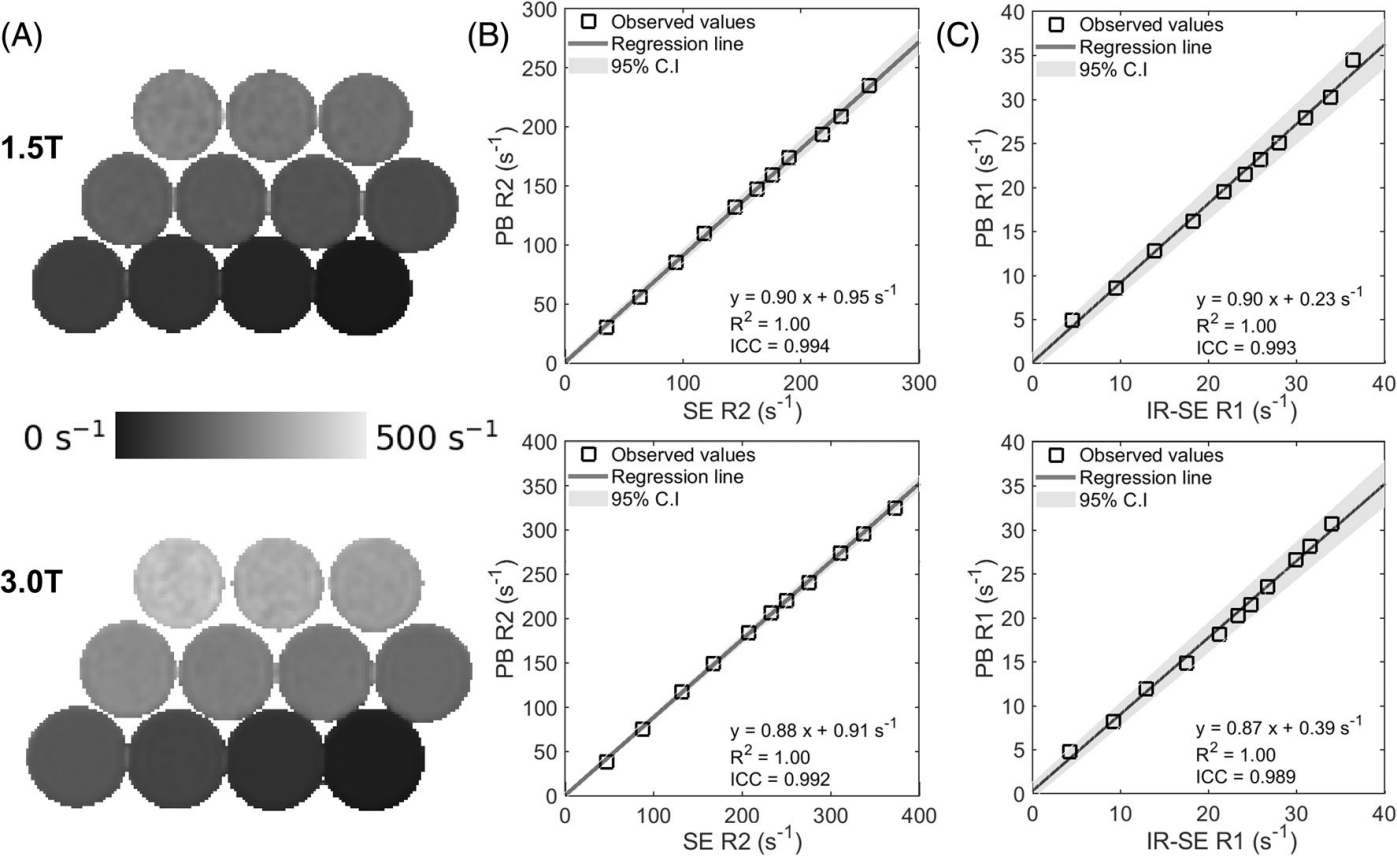
Phantom experiments demonstrate good agreement between the phase‐based R_2_ and reference spin‐echo (SE) R_2_ estimates. (A) Reference R_2_ map generated from a single‐echo SE sequence at both 1.5 T and 3.0 T. (B) Linear regression of phase‐based R_2_ as a function of single‐echo SE R_2_ is shown for both 1.5 T and 3.0 T. Slope and intercept are shown with their 95% confidence interval. (C) The PB R_1_ measurements also closely matched the IR‐SE R_1_ values, indicating the successful implementation of the R_1_‐R_2_ calibration.

After R_1_ correction, PB R_2_ estimation demonstrated close agreement with reference R_2_ estimates (Figure 4) at both 1.5 T (r^2^ = 0.99, slope = 0.90, intercept = 1.01.7 s^−1^) and 3.0 T (r^2^ = 0.99, slope = 0.9010.006, intercept = 0.911.5 s^−1^). Furthermore, PB R_1_ estimation closely matched the reference R_1_ values measured using the IR‐SE at both 1.5 T (r^2^ = 0.99, slope = 0.900.05, intercept = 1.01.1 s^−1^) and 3.0 T (r^2^ = 0.99, slope = 0.8740.004, intercept = 0.390.97 s^−1^).

### 4.6 In vivo feasibility study

**FIGURE 5:**
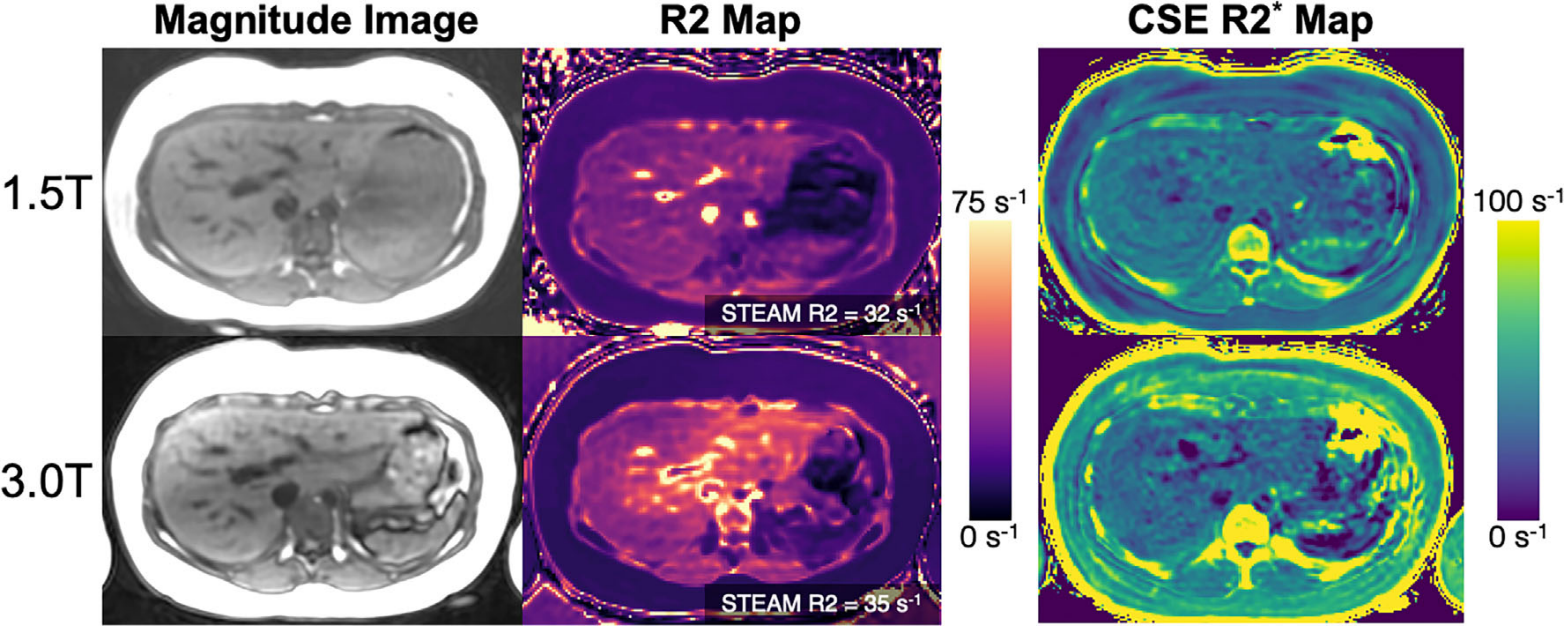
Volunteer experiments demonstrate good agreement between the phase‐based (PB) R_2_ and reference STEAM R_2_ estimates at both 1.5 T and 3.0 T. This is shown in examples of PB GRE magnitude images and R_2_ maps from a volunteer, with corresponding STEAM R_2_ estimates and R_2_* maps. To mitigate bias from noise, the spatial averaging approach was implemented by applying a spatial averaging (Subsection 3.2). The R_2_ maps were then generated using the R_1_‐R_2_ calibration method described in Subsection 3.2.

**FIGURE 6:**
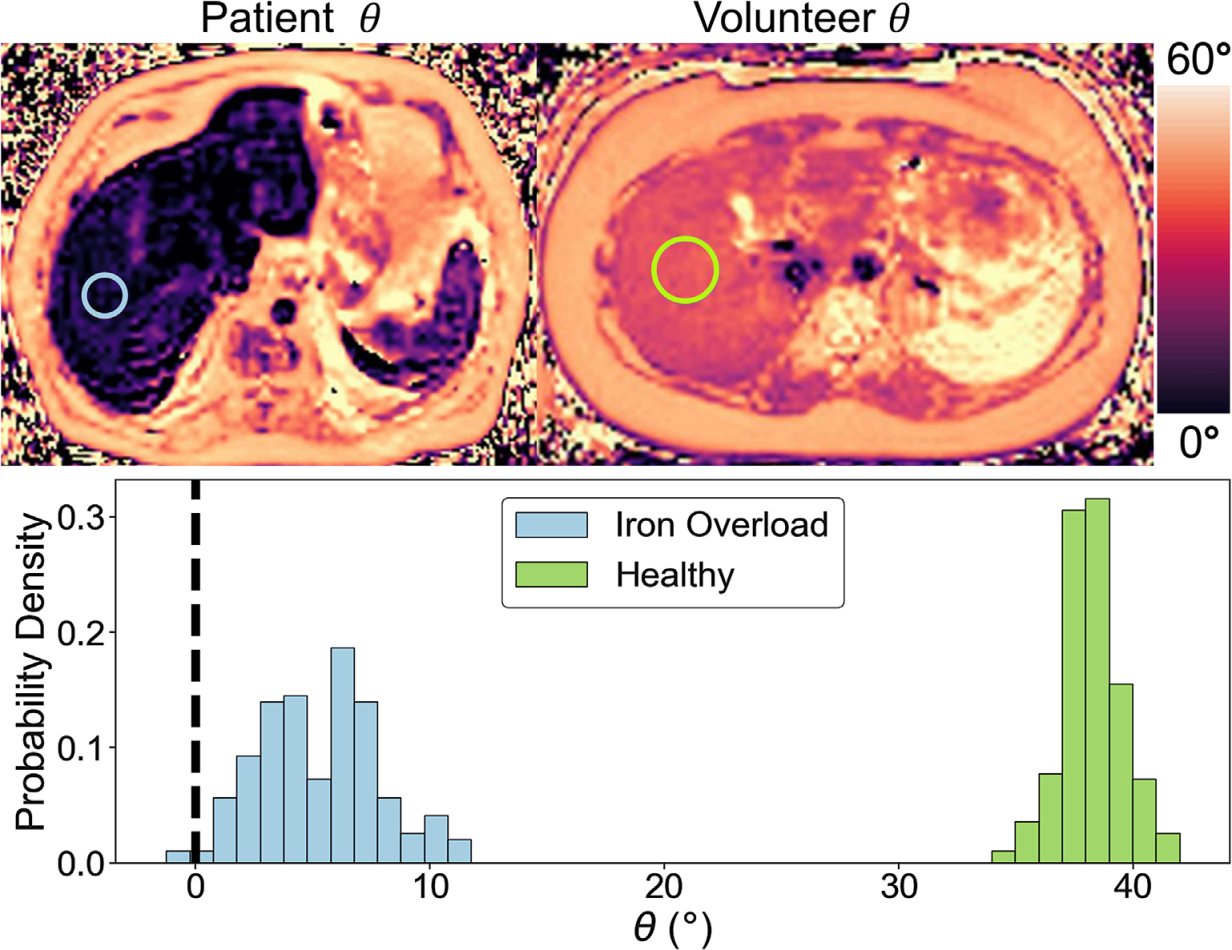
ROI histogram analysis comparing healthy and iron overloaded tissue demonstrates the necessity of the spatial averaging technique in high R2 tissue. An ROI (green) drawn in the GRE signal phase (θ) map of the healthy volunteer liver demonstrates high signal phase with no negative phase values. An ROI (blue) drawn in the phase map of a patient liver with iron overload has some voxels with negative phase values which map to inaccurate R2s if no averaging is done.

Good image quality was seen in the PB R_2_ of healthy volunteers generated from the PB R_2_ acquisition (Figure 5) after spatial averaging and R_1_ correction. The necessity of spatial averaging is demonstrated through the comparison of the phase distribution in a liver ROI between a healthy volunteer and a patient with severe iron overload (Figure 6). The phase in the iron overload patient phase is considerably lower than that from the healthy volunteer, with some negative phase values because of the effects of noise.

**FIGURE 7:**
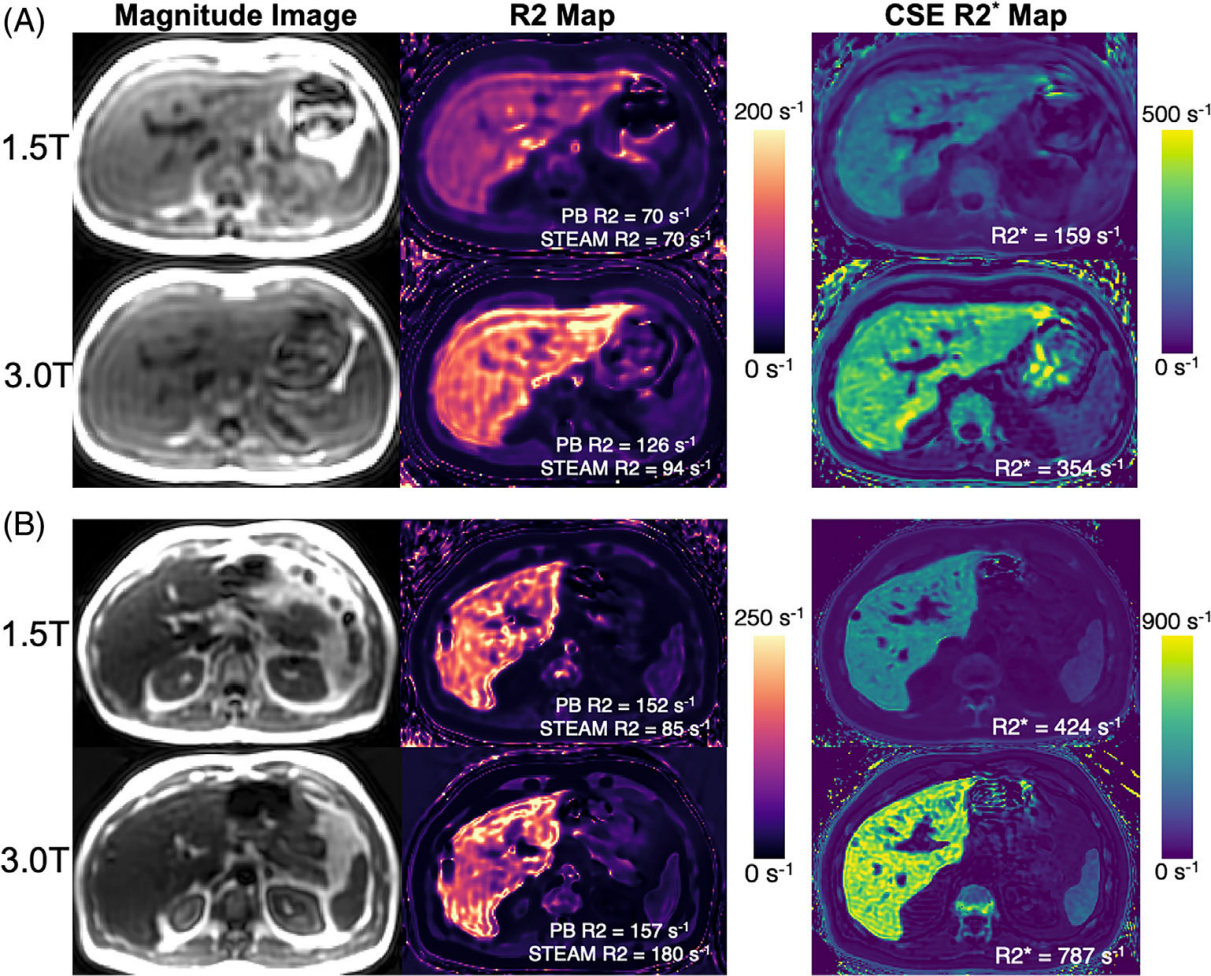
Studies in patients with iron overload demonstrate the ability of the phase‐based (PB) R_2_ method to quantify iron content in high R_2_ tissue. The magnitude and R_2_ maps exhibited good subjective image quality and apparent SNR, despite the high iron concentration in the liver. R_2_ could be biased because of the presence of motion artifacts. Reference STEAM R_2_ estimates are possibly underestimated in this patient because of very high iron levels affecting quality of R_2_ estimation (Simchick et al.^27^)

An example of PB R_2_ estimation in a patient with iron overload is shown in Figure 7. These R_2_ maps in have undergone spatial averaging and R_1_ correction. Good subjective image quality, including good apparent SNR, was seen in both magnitude and R_2_ maps, despite high iron concentration in the liver. In cases with high R_2_ values, an inhomogeneous distribution can be observed in the R_2_ map because of artifacts (Figure 7B). R_2_* maps generated from CSE‐MRI are also shown for comparison.

**FIGURE 8:**
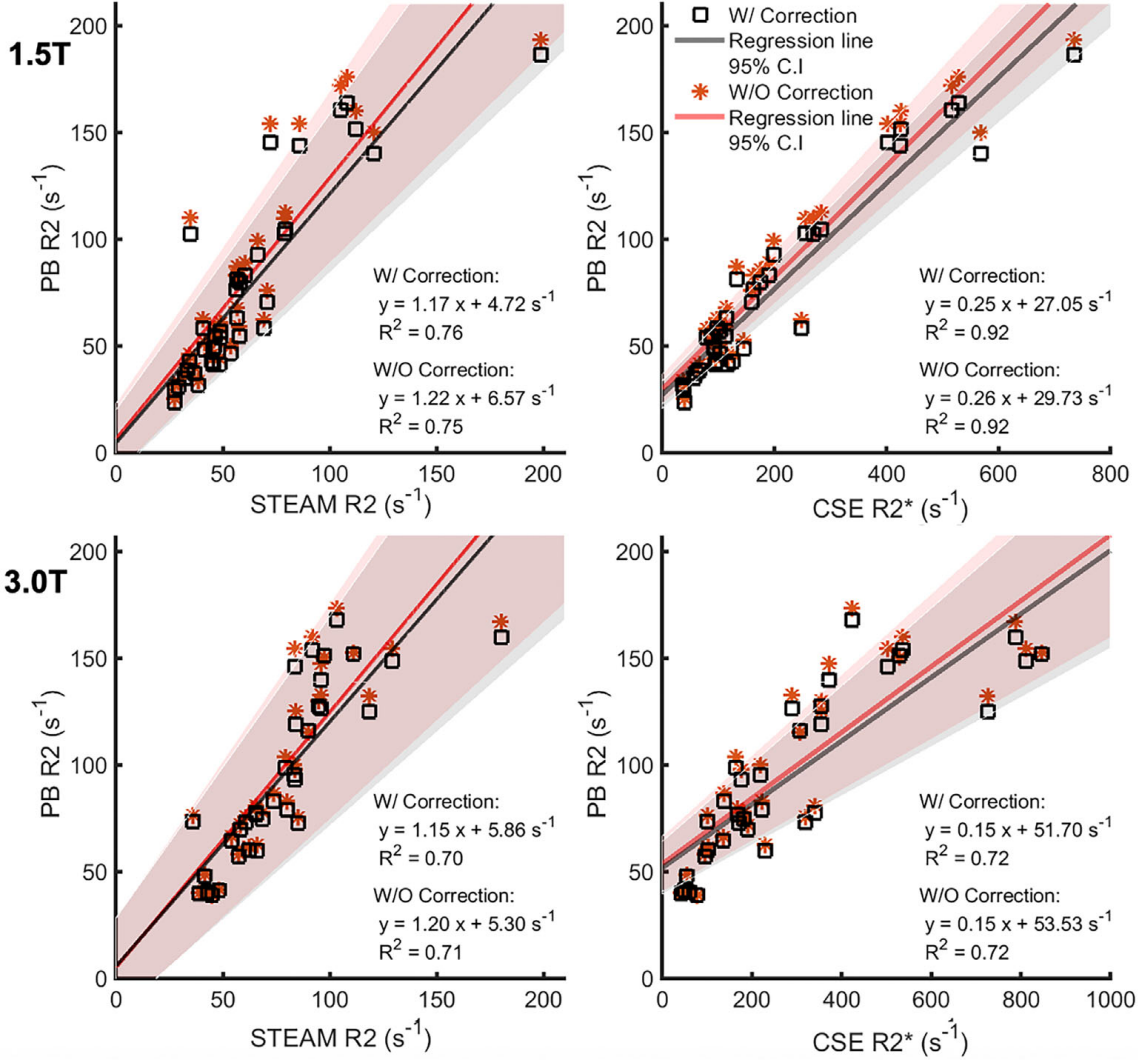
Phased‐based (PB) T_2_ agreed well with STEAM R_2_ and chemical shift‐encoded (CSE) R_2_*. There is a strong correlation between STEAM R_2_ and PB R_2_ at both 1.5 T and 3.0 T, although slight overestimation was observed. The plots show observed values, regression lines, and 95% confidence intervals. At 1.5 T, STEAM R_2_ versus PB R_2_ demonstrates strong correlation (r^2^ = 0.76, intraclass correlation coefficient [ICC] = 0.838), whereas CSE R_2_* versus PB R_2_ shows a higher correlation (r^2^ = 0.92, ICC = 0.465). At 3.0 T, STEAM R_2_ versus PB R_2_ maintains a similar correlation (r^2^ = 0.70, ICC = 0.794), and CSE R_2_* versus PB R_2_ shows moderate correlation (r^2^ = 0.72, ICC = 0.289). Without R_1_ correction and spatial averaging, R_2_ values were overestimated by approximately 4% (red regression lines), a statistically significant difference compared to those obtained with R_1_ correction and spatial averaging.

**FIGURE 9:**
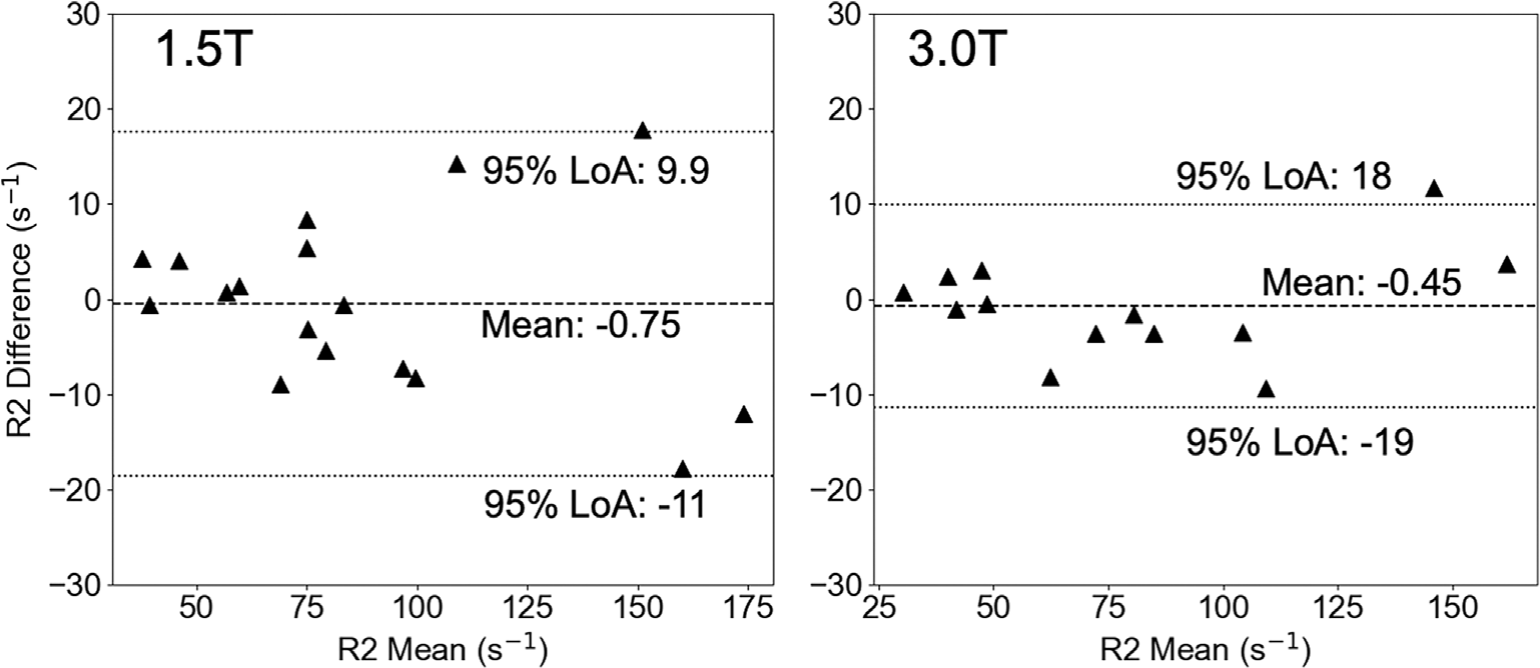
The Bland–Altman demonstrated excellent reproducibility of phase‐based (PB) R_2_ measurements across different field strengths. The plots demonstrate excellent reproducibility of the PB R_2_ measurements in iron overload patients, with no systematic bias observed, although some variability is noted at high R_2_ values at 3.0 T. Overall, these results confirm the reliability of the phase‐based R_2_ measurements for clinical use.

R_2_ estimation with proposed R_1_ correction and spatial averaging demonstrates close agreement between PB R_2_ and reference STEAM R_2_ across all healthy volunteers and iron‐overload patients (Figure 8) at 1.5 T (r^2^ = 0.76, slope = 1.17 ± 0.28, intercept = 4.72 ± 23 s^−1^) and 3.0 T (r^2^ = 0.70 ± 0.24, slope = 1.15, intercept = 5.86 ± 16 s^−1^), although PB R_2_ measurements tended to overestimate R_2_ compared to STEAM‐MRS. PB R_2_ also shows strong correlation with R_2_* measured using CSE‐MRI, which also indicates good sensitivity of PB R_2_ against iron deposition at both 1.5 T (r^2^ = 0.92, slope = 0.25 ± 0.027, intercept = −27 ± 6.9 s^−1^) and 3.0 T (r^2^ = 0.72 ± 0.035, slope = 0.15, intercept = 52 ± 13 s^−1^). In the absence of R_1_ correction and spatial averaging, R_2_ values were overestimated by approximately 4% compared to values obtained with R_1_ correction and spatial averaging, which is statistically significant (*p* < 0.001 at both 1.5 T and 3.0 T). Additionally, the results of linear regression analyses for R_2_ estimations obtained using only R_1_ correction and those obtained using only spatial averaging are presented in Figure S2. Bland–Altman analysis for the repeated RB R_2_ measurements demonstrated excellent repeatability at both 1.5 T (ICC = 0.991) and 3.0 T (ICC = 0.974) (Figure 9). PB R_1_ estimation in vivo agreed moderately with the reference STEAM R_1_ at both 1.5 T (R^2^ = 0.41, slope = 1.2 ± 0.53, intercept = −1.8 ± 0.96 s^−1^) and 3.0 T (R^2^ = 0.40, slope = 0.89 ± 0.66, intercept = 1.5 ± 0.39 s^−1^).

## 5 DISCUSSION AND CONCLUSIONS

In this work, we have developed and successfully demonstrated the feasibility of a modified PB R_2_ technique for whole‐liver volumetric R_2_ mapping in both phantoms and in iron overloaded patients, within a single breathhold, at both 1.5 T and 3.0 T. Despite the success of MRI methods such as SE R_2_ mapping for non‐invasive quantification of liver iron content, important limitations including long acquisition times and poor volumetric coverage remain. To address these limitations, we proposed the use of the PB R_2_ method for rapid, whole‐liver iron quantification. To enable R_2_ estimation in patients with iron overload, the following modifications were made to the original PB R_2_ method: the use of reduced TRs, spatial averaging, and R_1_ correction. With these modifications, the ability of this method to image high tissue across a wide range of R_2_ values was successfully demonstrated in phantoms, healthy volunteers, and patients with iron overload. As a result, the proposed PB R_2_ mapping technique may enable screening at‐risk populations and monitoring of patients undergoing treatment for iron overload.

The proposed method may have important implications for the non‐invasive assessment of LIC. Accurate and precise liver iron quantification is necessary for the detection and treatment of patients with iron overload. Fundamental limitations such as lengthy acquisition times, poor volumetric coverage, and limited performance at high iron concentrations remain a barrier for clinical use of current R_2_ mapping methods. In our previous study, although we demonstrated the feasibility of rapid and 3D R_2_ mapping in the liver, a relatively long TR of 6.5 ms was used, that limits the dynamic range of R_2_ up to approximately 100 s^−1^, corresponding to approximately 7 mg/g at 1.5 T and 4 mg/g at 3.0 T. The modified PB R_2_ method has the benefit of full volumetric coverage of the liver within a single breathhold and accurately quantifies R_2_ in even severe iron overload patients at both 1.5 T and 3.0 T, potentially facilitating its clinical use. As noted in the Theory section, the theoretical maximum measurable LIC with this method is approximately 32 mg/g at 1.5 T and 15 mg/g at 3.0 T. This may be further increased with increasingly higher‐performance MR systems or reducing the resolution to achieve shorter TRs. The total acquisition time was approximately 11 s, easily acquired with a single breathhold, allowing for imaging even in patients who may have trouble performing a longer breathhold, such as children.

Wang et al.^17^ first proposed and successfully demonstrated feasibility of the PB T_2_/R_2_ mapping method for rapid, whole‐liver R_2_ quantification in healthy volunteers at 3.0 T. Although this method works reliably in tissues with normal iron concentration, a new strategy was necessary for quantification of R_2_ in the setting of elevated iron concentration. Very short TR is necessary to increase the maximum R_2_ measurable and spatial averaging is necessary to avoid non‐physical negative phase values, and estimation bias of R_2_, because of noise. In addition, R_1_ correction is necessary to account for increases in R_1_ in the presence of elevated iron concentrations. The proposed method with these modifications allows for accurate R_2_ quantification in even high iron concentrations. Notably, the method described in this work also removes the need for a priori knowledge of the R_1_ for the lookup table, initially proposed by Wang et al.^17^ Indeed, the proposed method provides an estimate of tissue R_1_, although this is beyond the scope of this work and further research would be needed to explore the use of this approach to estimate R_1_.

The main limitations of this study are the limited patient population size and use of only one MR system vendor. Further studies in a larger patient cohort and multi‐center, multi‐vendor analysis are needed. Another limitation is that the R_2_ measurement could be significantly biased in patients with concomitant fat and iron accumulation, although this is also a limitation of current SE‐based R_2_ mapping methods^38^ that do not correct for the presence of fat. However, emerging fat suppression techniques, such as short water‐excitation pulses^39^, ^40^, ^41^ may help mitigate this limitation, at least at moderate iron concentrations when water and fat resonance peaks are still distinct.

Some motion artifacts originating from subcutaneous fat in the posterior abdomen were observed in several patients, leading to non‐negligible phase errors. Using water‐only excitation may help mitigate potential bias from motion related ghosting of fat signal from adipose tissue. B_1_ inhomogeneity is also a potential source of error in R_2_ estimation, especially at 3.0 T, given a non‐zero, albeit weak dependence of the lookup table on flip angle. In this study, flip angle and phase increments were chosen to minimize this effect in the liver. Future work should examine the need for B_1_ correction and potential correction strategies, especially at higher field strengths such as 3.0 T.

Another limitation is that a long TE in a GRE sequence could lead to reduced signal because of T_2_* decay in the presence of severe iron overload. However, we note that the TE with the proposed method was approximately 1 ms for our studies. If we assume a tissue T2* of approximately 1ms, corresponding to severe iron overload, this will mean that approximately 1⁄e≈37% of the signal remains after one TE, which should be sufficient to reliably measure the phase of MR signal, and perform subsequent PB R2 estimation.

The proposed spatial averaging strategy offers several advantages over simply acquiring lower‐resolution images. It allows for post‐acquisition flexibility in adjusting the kernel size for averaging. This adaptability enables researchers to optimize the trade‐off between spatial resolution and SNR based on the specific requirements of each dataset. For instance, if the SNR of the R_2_ map is sufficient, the native resolution can be maintained. Conversely, in scenarios where noise is a significant concern, particularly with high R_2_ values, a larger kernel size can be used to obtain an unbiased R_2_ map with improved SNR. This approach provides a versatile method for balancing spatial detail and measurement accuracy in R_2_ mapping, tailored to the characteristics of individual datasets. The proposed spatial averaging is important to avoid negative phase artifacts. However, spatial averaging also reduces spatial resolution, which may affect R_2_ measurements in spatially heterogeneous regions (e.g., near blood vessels). After applying spatial averaging with a rectangular filter with kernel size of 3 × 3, the effective in‐plane resolution was 12 × 12 mm^2^, which is approximately 2.5 times larger than the original voxel size. Although this resolution is insufficient to distinguish small abnormal tissues, it is adequate for excluding major blood vessels (with approximate diameter 15 mm^42^), which could otherwise affect R_2_ measurements. An evaluation of varying filter kernel sizes is included in Figure S3.

R_2_ estimation in blood vessels may be inaccurate because of flow effects. PB R_2_ imaging preserves the transverse magnetization; therefore, phase errors accumulate from one TR to the next because of unbalanced gradient moments. As a result, the R_2_ estimation may be biased from the accumulated phase errors because of moving blood. Additionally, PB R_2_ imaging uses a smaller RF phase increment (˜1–2°) compared to typical spoiled GRE sequences (˜117°), resulting in partial spoiling. This partial spoiling has been shown to suppress signals from tissues with long T_2_.^43^ Because blood has a relatively long T_2_ (>200 ms^44^), the signal magnitude from blood is suppressed, which could lead to additional R_2_ bias in blood vessels because of low SNR.

The relationship between R_1_ and R_2_, which is crucial for accurate R_1_ correction, needs to be evaluated carefully. Although we have successfully demonstrated the concept of R_1_ correction using a phantom model, the R_1_‐R_2_ relationship in the phantom is different in liver tissue. This difference could lead to different noise properties compared to those in the liver. Furthermore, the R_1_‐R_2_ relationship used for the liver was obtained from a previous study in patients with liver disease, and may not accurately represent the current study population. Therefore, although the phantom experiments convincingly demonstrate the R_1_ correction concept, they are insufficient to fully validate the accuracy of the proposed method. It will be important to conduct further studies in patients to enable direct validation of the proposed R_1_ correction in the liver.

In summary, we have proposed and successfully demonstrated the feasibility of a modified PB R_2_ method for rapid whole‐liver iron quantification within a breathhold, through optimization simulations, phantom experiments, healthy volunteer studies, and iron overload patient studies at both 1.5 T and 3.0 T. This approach has the potential for rapid, non‐invasive, whole‐liver assessment of iron over a wide range of LICs, as needed for the accurate detection and treatment monitoring of iron overload.

## Supporting information


**FIGURE S1.** The bias and CV both decrease as the TR decreases. (a) The plot of bias as a function of SNR for TR = 3, 6, and 9 ms shows that bias increases significantly with higher R2 values due to decreased SNR. Shorter TRs (e.g., 3 ms) result in lower bias across all SNR levels. (b) The plot of CV shows that it follows a similar trend to bias, increasing with higher R2 values. The CV is markedly higher at R2 = 300 s^−1^, even when SNR is high, highlighting the challenges in achieving precise measurements at high R2 values. The use of shorter TRs reduces CV across all SNR levels, emphasizing the benefit of reduced TR in improving measurement accuracy.
**FIGURE S2.** R1 correction primarily contributes to reducing the overestimation of R2 values. The slopes of the regression lines for R2 values obtained with R1 correction were similar to those obtained when both R1 correction and spatial averaging were applied (see Figure 8), indicating that spatial averaging adds minimal benefit once R1 correction is implemented. In contrast, the regression lines for R2 values obtained using only spatial averaging still showed notable overestimation, emphasizing that R1 correction is essential for accurate R2 quantification in the protocol we used.
**FIGURE S3.** As the kernel size for spatial averaging increases, blurring becomes significant. When the kernel size is much larger than the vessel size (approximately 15 mm), the boundaries become unclear, which could impact R2 measurements due to partial volume effects.

## Data Availability

Phantom, volunteer human data, and MATLAB code will be made available at (https://github.com/dtamadauw/PhaseBasedR2MappingLiver/).
