# Supplementary material for: Whole liver phase‐based R2 mapping in liver iron overload within a breath‐hold
Source: Magn Reson Med. 2025 Feb 18;94(1):183–98. doi: 10.1002/mrm.30461 (PMC12021320; doi:10.1002/mrm.30461)
Supplement: Supplementary file 1 — FIGURE S1. The bias and CV both decrease as the TR decreases. (a) The plot of bias as a function of SNR for TR = 3, 6, and 9 ms shows that bias increases significantly with higher R2 values due to decreased SNR. Shorter TRs (e.g., 3 ms) result in lower bias across all SNR levels. (b) The plot of CV shows that it follows a similar trend to bias, increasing with higher R2 values. The CV is markedly higher at R2 = 300 s−1, even when SNR is high, highlighting the challenges in achieving precise measurements at high R2 values. The use of shorter TRs reduces CV across all SNR levels, emphasizing the benefit of reduced TR in improving measurement accuracy. FIGURE S2. R1 correction primarily contributes to reducing the overestimation of R2 values. The slopes of the regression lines for R2 values obtained with R1 correction were similar to those obtained when both R1 correction and spatial averaging were applied (see Figure 8), indicating that spatial averaging adds minimal benefit once R1 correction is implemented. In contrast, the regression lines for R2 values obtained using only spatial averaging still showed notable overestimation, emphasizing that R1 correction is essential for accurate R2 quantification in the protocol we used. FIGURE S3. As the kernel size for spatial averaging increases, blurring becomes significant. When the kernel size is much larger than the vessel size (approximately 15 mm), the boundaries become unclear, which could impact R2 measurements due to partial volume effects. [file MRM-94-183-s001.docx]

**Supporting Information**

Whole Liver Phase-Based R2 Mapping in Liver Iron Overload within a Breath-hold

Daiki Tamada, Jayse Weaver, Rianne van der Heijden, Diego Hernando, Scott B Reeder

**APPENDIX S1**

Monte Carlo simulations were performed to investigate measurement bias and variability with varying repetition times (TRs). Signals were calculated using the Bloch equation with the parameters described in Section 3.4. Gaussian noise was added to the calculated signals to achieve signal-to-noise ratios (SNR) ranging from 5 to 50. The simulations were conducted with R2 values of 30, 150, and 300 s^-1^. For each pair of TR and SNR, 10000 signals were calculated. The bias of the signal was calculated as $100\times\frac{({R^{'}}_{2}-R2)}{R_{2}}$, where R2 and R2’ are true and estimated R2 values, respectively. Also, coefficient variance (CV) was calculated as $100\times\frac{\sigma}{R_{2}}$, where $\sigma$ is standard deviation of the R2 estimation.

As shown in **Figure S1**, both bias and CV are decrease as TR decreases. In the case of R2 of 30 s^-1^, even longer TRs can be estimate R2 with less than 5% of bias when SNR is above 10. The results also show that a TR of 3 ms, which is an optimized TR, provides smallest bias and CV, indicating that the optimized TR offers better sensitivity for both normal liver and iron-overloaded liver. However, the bias exceeds 80% for R2 of 300 s^-1^ even with high SNR because of severe signal decay. Using shorter TRs helps to decrease bias and CV across all SNR levels, highlighting the advantage of employing a reduced TR to enhance measurement accuracy.


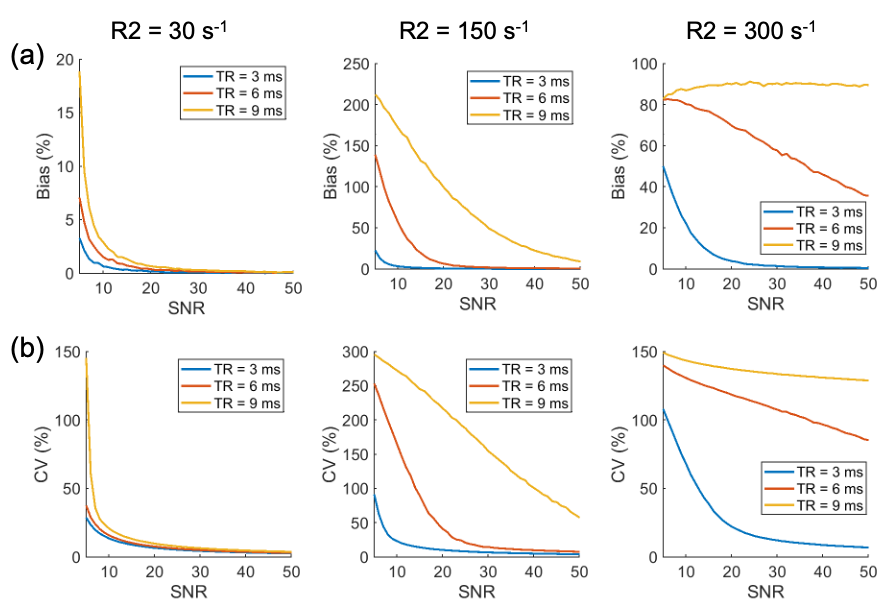


**Supporting Information Figure S1** The bias and coefficient of variation (CV) both decrease as the TR decreases. (a) The plot of bias as a function of SNR for TR = 3 ms, 6 ms, and 9 ms shows that bias increases significantly with higher R2 values due to decreased SNR. Shorter TRs (e.g., 3 ms) result in lower bias across all SNR levels. (b) The plot of CV shows that it follows a similar trend to bias, increasing with higher R2 values. The CV is markedly higher at R2 = 300 s⁻¹, even when SNR is high, highlighting the challenges in achieving precise measurements at high R2 values. The use of shorter TRs reduces CV across all SNR levels, emphasizing the benefit of reduced TR in improving measurement accuracy.

**APPENDIX S2**

To demonstrate the effect of R1 correction and spatial average filtering on R2 estimation, we performed linear regression analyses for R2 values obtained using only R1 correction and those obtained using only spatial average filtering. The same regions of interest (ROIs) as explained in subsection 3.9 were used for R2 measurements. The slopes of the regression lines for R2 values obtained with R1 correction were similar to those obtained with both R1 correction and spatial average filtering. In contrast, the regression lines for R2 values obtained using only spatial average filtering still showed overestimation. These results indicate that R1 correction primarily contributed to reducing the overestimation of R2. However, spatial average filtering could be essential when SNR is low.


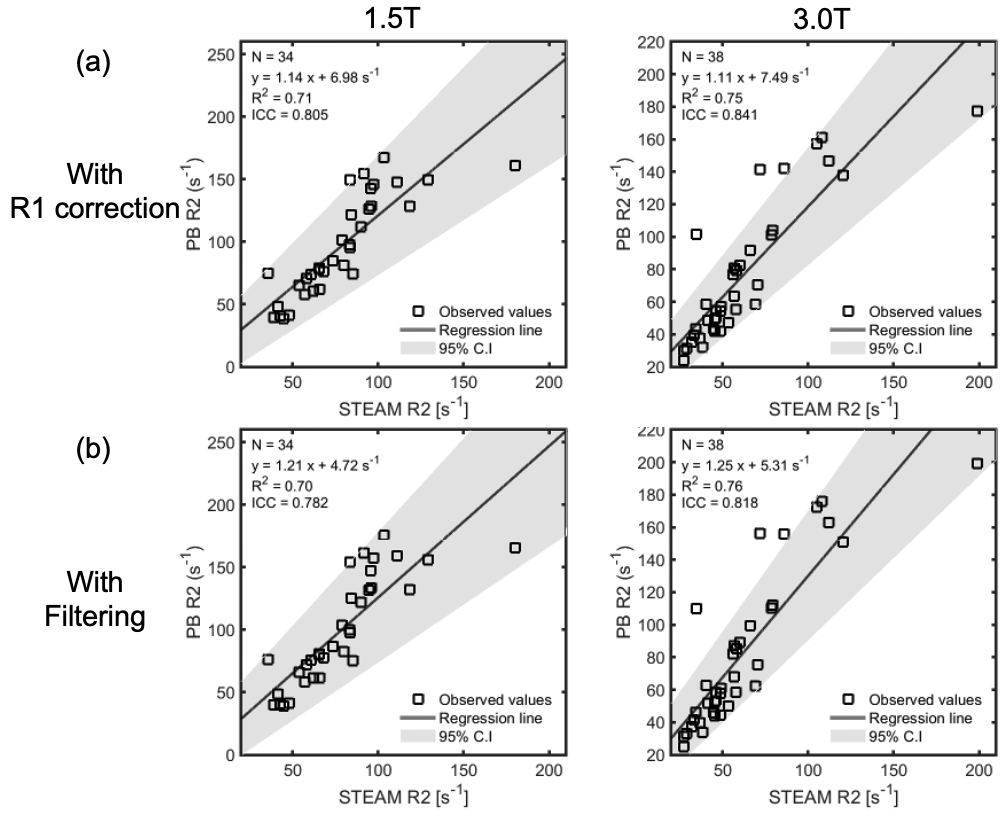


**Supporting Information Figure S2** R1 correction primarily contributes to reducing the overestimation of R2 values. The slopes of the regression lines for R2 values obtained with R1 correction were similar to those obtained when both R1 correction and spatial average filtering were applied (see Figure 8), indicating that spatial averaging adds minimal benefit once R1 correction is implemented. In contrast, the regression lines for R2 values obtained using only spatial average filtering still showed notable overestimation, emphasizing that R1 correction is essential for accurate R2 quantification in the protocol we used.

**APPENDIX S3**

To demonstrate the effect of spatial average filtering, we applied it using different kernel sizes. As mentioned in Discussion, the resolution of R2 maps decreases as the kernel size increases. Using kernel sizes ranging from 1×1 to 5×5, corresponding to resolutions of 4.8×4.8 mm² to 22×22 mm², we were able to distinguish the liver and vessels. However, if the kernel size is much larger than the vessel size, the boundaries become unclear, which may lead to bias in R2 measurements due to the partial volume effect.


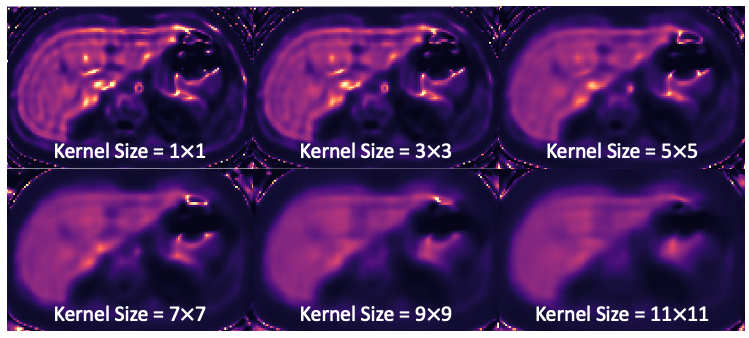


**Supporting Information Figure S3** As the kernel size for spatial average filtering increases, blurring becomes significant. When the kernel size is much larger than the vessel size (approximately 15 mm), the boundaries become unclear, which could impact R2 measurements due to partial volume effects.
